# Photoacoustic probes for real-time tracking of endogenous H_2_S in living mice†
†Electronic supplementary information (ESI) available: Procedures for the synthesis of BODPA, living mice imaging, characterization data, and supplementary figures. See DOI: 10.1039/c6sc04703c



**DOI:** 10.1039/c6sc04703c

**Published:** 2016-11-30

**Authors:** Ben Shi, Xianfeng Gu, Qiang Fei, Chunchang Zhao

**Affiliations:** a Key Laboratory for Advanced Materials , Institute of Fine Chemicals , School of Chemistry and Molecular Engineering , East China University of Science & Technology , Shanghai 200237 , P. R. China . Email: zhaocchang@ecust.edu.cn; b Department of Medicinal Chemistry , School of Pharmacy , Fudan University , Shanghai , 201203 China

## Abstract

H_2_S is a key chemical mediator that exerts a vital role in diverse physiological and pathological processes. However, *in vivo* tracking of endogenous H_2_S generation still remains difficult due to the lack of reliable analytical methods. Herein, we present the first example of activatable photoacoustic probes for real-time imaging of H_2_S in living mice through the full utilization of the superiority of photoacoustic imaging modality at fine spatial resolution during deep tissue penetration. The designed probe can generate high NIR absorption at 780 nm in the presence of H_2_S, thus producing a strong photoacoustic signal output in the NIR region. Furthermore, this probe exhibits extremely fast and highly selective responsiveness, good water-solubility and excellent biocompatibility. In light of these outstanding features, this probe realizes the direct photoacoustic trapping of endogenous H_2_S generation in a HCT116 tumor-bearing mouse model. These preliminary imaging studies show that HCT116 colon tumors exhibit CBS upregulation activity, resulting in an increased rate of H_2_S generation.

## Abstract 2

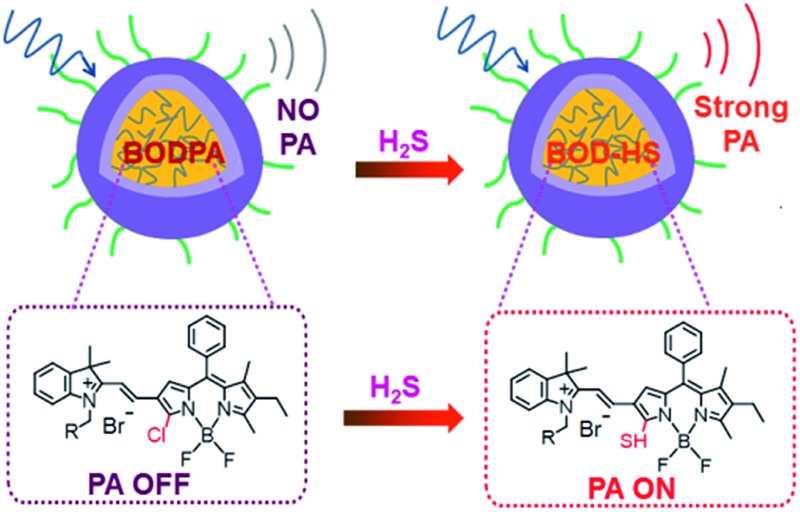
H_2_S-activatable probes showed an extremely fast and highly selective photoacoustic response to H_2_S, permitting real-time photoacoustic trapping in living mice.

## Introduction

Mounting studies have shown that H_2_S plays a vital role in diverse physiological and pathological processes. It has been identified as a physiological gasotransmitter with robust cytoprotective actions in multiple organ systems, including regulatory roles in the cardiovascular system and modulation of the central nervous, respiratory, and gastrointestinal systems.^1,2^ Nevertheless, abnormal production of H_2_S can contribute to a variety of diseases ranging from Down's syndrome and Alzheimer's disease to diabetes and hypertension.^3^ As such, it is of great scientific interest to develop selective methods for the real-time tracking of this small molecule in living systems. To data, a number of reaction-based fluorescent probes have been designed as attractive molecular imaging techniques for monitoring H_2_S in living cells.^4–7^ However, few probes have been employed for *in vivo* H_2_S imaging due to the fact that fluorescence imaging suffers from limitations such as shallow tissue penetration and poor spatial resolution inside deep biological tissue. Therefore, development of reaction-based probes with new imaging modality is highly demanded in order to address these challenges.

Photoacoustic (PA) imaging is a state-of-the-art imaging modality which relies on the translation of excitation light into ultrasonic waves based on the PA effect, and provides deeper tissue imaging penetration and higher *in vivo* spatial resolution when compared with traditional optical imaging techniques.^8^ As a result of these significant advantages, PA imaging has found broad applications in biology and medicine as a noninvasive imaging tool.^9,10^ However, it is still a big challenge to devise activatable probes for the photoacoustic imaging of molecular targets of interest. Given that H_2_S is a key chemical mediator, the full utilization of the superiority of PA imaging modality for the construction of H_2_S-activatable photoacoustic probes should be imperative to fully explore the biological roles of H_2_S *in vivo*. To our knowledge, there are no probes available for the PA imaging of this important small molecule. Herein, we present the first example of reaction-based PA probes that show a fast response to H_2_S accompanied with “turn-on” PA signals.

**Scheme 1:**
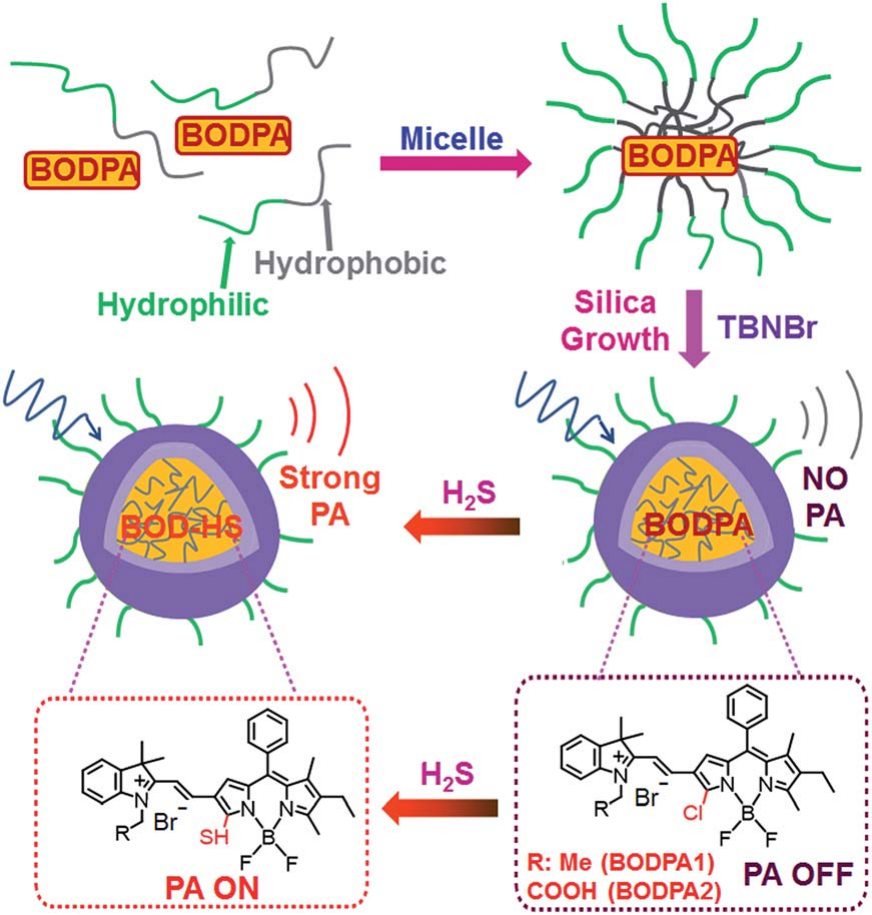
Schematic illustration of the construction of activatable photoacoustic probes for H_2_S.

In this contribution, a H_2_S-activatable probe was fabricated by encapsulating semi-cyanine-BODIPY hybrid dyes (BODPA) into the hydrophobic interior of core–shell silica nanocomposites for *in vivo* photoacoustic imaging (Scheme 1). Our design strategy is based on modulating the electronic nature of the substituents to generate a near-infrared absorbing dye to produce PA signals, employing the feasible thiol–halogen nucleophilic substitution of a monochlorinated hybrid dye with H_2_S. Indeed, the presence of H_2_S transformed BODPA into BOD-HS inside nanoparticles through aromatic nucleophilic substitution, which resulted in high NIR absorption around 780 nm. Thus, the Si@BODPA probe generated a strong photoacoustic signal output in the NIR region. As shells of these nanoparticles were composed of poly(ethylene glycol) (PEG) and positively charged silica units, Si@BODPA possessed good water-solubility and excellent biocompatibility as well as extremely fast responsiveness, enabling real-time imaging of H_2_S-related biological processes. In light of these outstanding features, Si@BODPA allowed the direct photoacoustic tracking of endogenous H_2_S generation in a HCT116 (human colon cancer cells) tumor-bearing mouse model.

## Results and discussion

Two monochlorinated BODIPY-based hybrid dyes (BODPA1 and BODPA2) were chosen to prepare core–shell silica nanocomposites. Both dyes responded to H_2_S readily and were efficiently converted to BOD-HS within 50 minutes in CH_3_CN/PBS buffer mixtures (1 : 1, v/v, 20 mM, pH 7.4, room temperature), which was demonstrated by HPLC and HRMS (Fig. S1†). Specifically, a strong NIR absorption band around 735 nm was activated, accompanied by a decrease of absorption at 540 nm (Fig. S2†), indicative of BODPAs as building blocks for the construction of activatable photoacoustic probes. Notably, BOD-HS is non-fluorescent which facilitates thermal deactivation to generate much stronger PA signals.^10*b*^ Considering the limited solubility of BODPAs as well as their slow reaction with H_2_S in aqueous solution, we here intended to apply hydrophilic nanoparticles for the encapsulation of BODPAs to overcome this issue. Firstly, BODPAs and mPEG-DSPE were mixed in a 0.85 N hydrochloride solution under rigorous stirring, leading to the formation of self-assembled micelles with hydrophobic inner cores and hydrophilic PEG chains as outer arms.^11^ The hydrophobicity of BODIPY dyes trap them in the interior core. Following the *in situ* growth of silica shells under acidic conditions with *N*-trimethoxysilylpropyl-*N*,*N*,*N*-tri-*n*-butylammonium bromide (TBNBr) as silica agents for cross-linking, water-dispersible nanoparticles (Si@BODPA) were afforded, which were dialyzed for 48 h to remove unreacted chemicals. Additional characterization using TEM and DLS were carried out, which were identified in Fig. S3.†

**Fig. 1:**
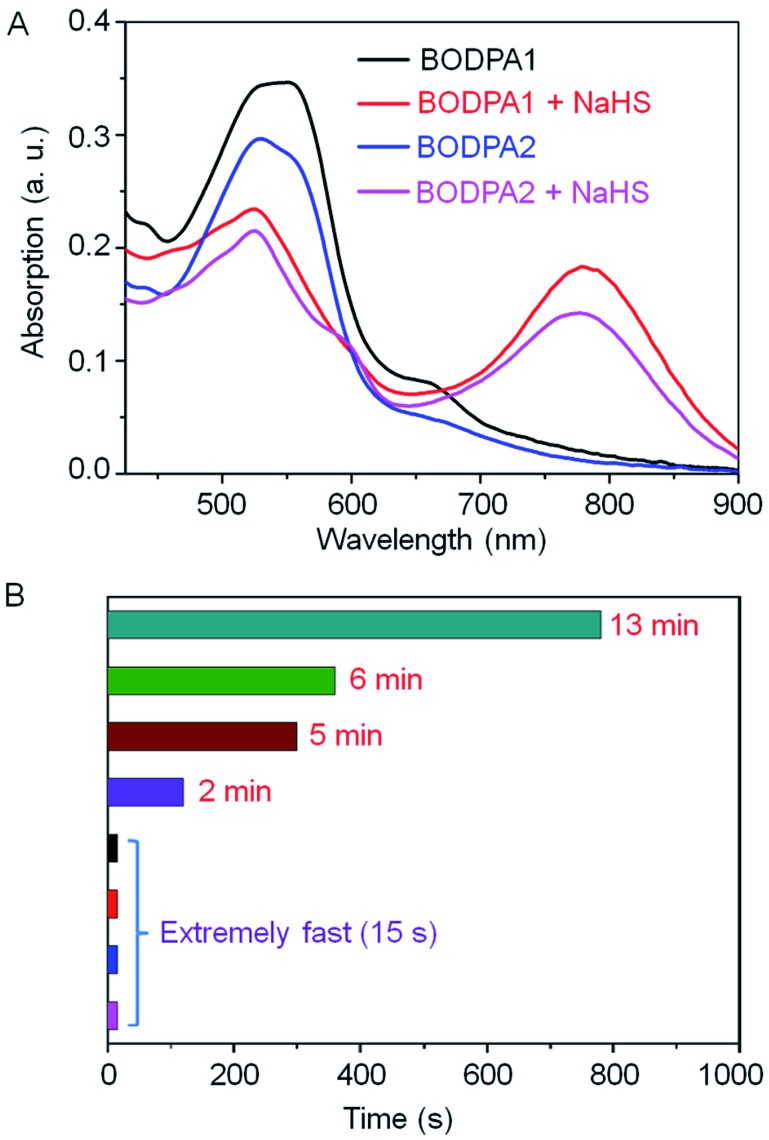
(A) The absorption changes of Si@BODPAs (10 μM BODPA1 or BODPA2) in the absence and presence of NaHS (100 μM). (B) The dynamics of the reaction between BODPAs (10 μM) and H_2_S (100 μM) in Si@BODPAs (13 min and 6 min for BODPA2 in Si@BODPA30 and Si@BODPA90, respectively; 5 min and 2 min for BODPA1 in Si@BODPA30 and Si@BODPA90, respectively. Si@BODPA180 and Si@BODPA270 afforded rapid detection of H_2_S within 15 s).

The response of Si@BODPA toward H_2_S was then evaluated in PBS buffer solutions (pH 7.4) using UV-vis spectroscopy. The addition of NaHS induced the generation of the NIR absorption at 780 nm and 777 nm for BODPA1 and BODPA2, respectively. The reduction of the original peak at around 540 nm was also noted (Fig. 1A). This observation was in good agreement with that of BODPAs when interacted with NaHS in CH_3_CN/PBS, which demonstrated the occurrence of a reaction between BODPAs and H_2_S within the water-dispersible silica core–shell nanoparticles. Notably, BOD-HS in the hydrophobic interior of the nanocomposites showed an obvious red-shift of 45 nm in its absorption compared to that in CH_3_CN/PBS buffer mixtures (1 : 1, v/v). This variation could be rationalized as the effect of H-bonding interactions between the sulfur atom and H atom of H_2_O. Such interactions existing in aqueous solutions rather than in nanocomposites reduced the electron-donating ability of sulfur which ultimately led to the shift of the absorption to shorter wavelengths. Impressively, the dynamics of the reaction between BODPAs and H_2_S in Si@BODPA was Si dose-dependent (Fig. 1B and S4†). In general, increasing the amount of Si in the shell, as determined by energy dispersive X-ray (EDX) spectroscopy (Fig. S5†), caused a much faster reaction. The process of absorption change in Si@BODPA30 could reach completion in about 5 and 13 minutes for BODPA1 and BODPA2, respectively. In the case of Si@BODPA90, such change was promoted to be about 2 min and 6 min for BODPA1 and BODPA2, respectively. Gratifyingly, the responsive reaction was dramatically accelerated when further increasing the Si content, resulting in ideal probes, Si@BODPA180 and Si@BODPA270, which could rapidly detect H_2_S within 15 s. Given the transient nature of H_2_S which is catabolized rapidly in biological systems, one of the most important prerequisites for a probe is the extremely fast reactivity toward H_2_S. Thus, the nanoprobes established here possess the superiority and capability of trapping transient H_2_S and monitoring of H_2_S-related biological processes in real-time. As an example, Si@BODPA180 was chosen as a model probe for studying its potential applications. Undoubtedly, the entrapment of BODPAs into the interior of silica nanocomposites enables the advantage of rapid reaction, which can be attributed to the transfer of H_2_S *via* the hydrophilic assembly from water into its polar interior, which facilitates nucleophilic substitution.^12^ Such a polar microenvironment within the core is due to the positively charged ammonium functionality in TBNBr. More importantly, these nanoparticles are smaller than 100 nm and therefore are feasibly endocytosed by cells.

**Fig. 2:**
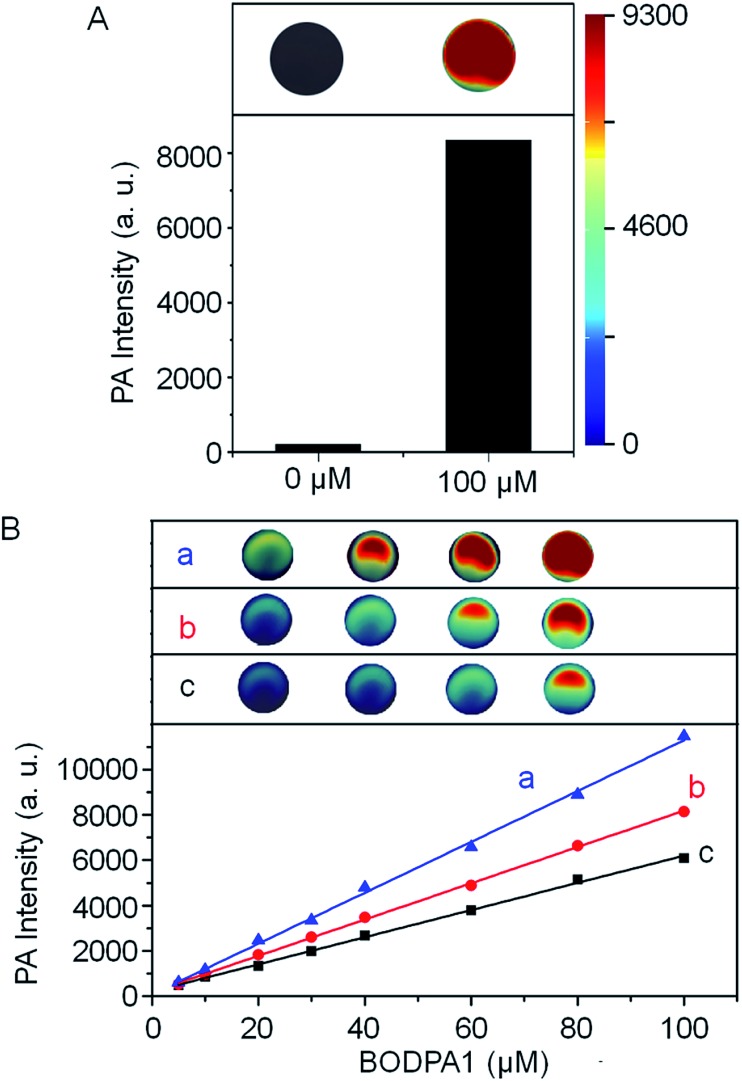
(A) Representative photoacoustic images and amplitudes of Si@BODPA180 (80 μM BODPA1) in the absence and presence of NaHS (100 μM). (B) Representative photoacoustic images and amplitudes of Si@BODPA180 as a function of BODPA1 concentration upon addition of two molar equivalents of H_2_S. (a): Si@BODPA180(7); (b): Si@BODPA180(4); (c): Si@BODPA180(1); these nanoparticles were obtained by employing the weight ratio (BODPA1/mPEG-DSPE) of 7/100, 4/100 and 1/100, respectively.

The photoacoustic responsiveness to H_2_S was then established. In the absence of H_2_S, Si@BODPA180 showed no detectable photoacoustic signals in PBS buffer solutions (pH 7.4). When H_2_S was introduced to the solution, Si@BODPA180 efficiently produced bright photoacoustic signals following excitation at 780 nm and ultimately led to a 44-fold turn-on response (Fig. 2A), which was in agreement with H_2_S-triggered formation of BOD-HS within nanoparticles. These results infer that this nanoprobe could serve as an activatable photoacoustic probe.

H_2_S triggered photoacoustic amplitudes of the nanocomposites upon excitation at 780 nm were examined at various concentrations. A linear correlation could be obtained between the PA signal and concentration (Fig. 2B). To explore the effect of local concentrations of BODPA within the interior of the nanoparticles on the activated PA properties, we also prepared Si@BODPA with different ratios of BODPA/mPEG-DSPE in weight. It was found that, at the same concentration of BODPA, nanoparticles Si@BODPA180(7) with the ratio of 7/100 exhibited higher PA signals than those of Si@BODPA180(1) and Si@BODPA180(4) obtained from the ratio of 1/100 and 4/100, inferring that Si@BODPA180(7) is a superior system for the establishment of photoacoustic imaging probes. These results reflect that elevated local concentrations of BODPA benefit the thermal deactivation of the excited chromophores, thereby affording stronger PA signals.

**Fig. 3:**
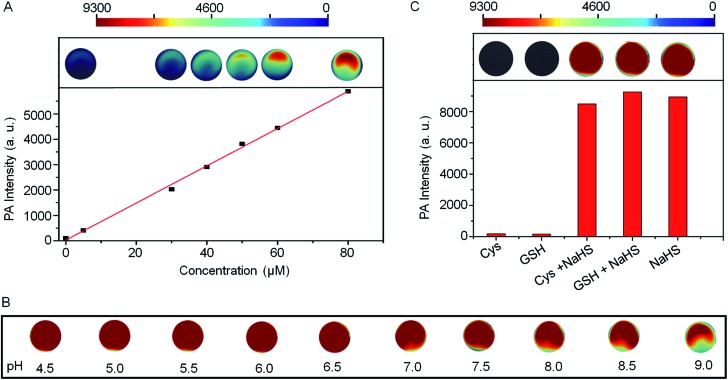
(A) The linear plot of PA intensities of Si@BODPA180(7) (80 μM BODPA1) as a function of H_2_S concentrations. (B) The pH effects on the PA responsive process of Si@BODPA180(7) toward H_2_S. (C) Photoacoustic amplitude changes of Si@BODPA180(7) (80 μM BODPA1) in the presence of NaHS and other biologically relevant competing analytes in PBS (pH 7.4) at room temperature. Data shown are for 1 mM glutathione, 1 mM cysteine, and 100 μM NaHS.

The PA intensities of Si@BODPA180(7) showed a linear and dose-dependent correlation with the concentrations of NaHS in the range of 0–80 μM (Fig. 3A). The detection limit was determined to be as low as 53 nM, suggesting sufficient sensitivity to detect endogenous H_2_S in living systems. Furthermore, Si@BODPA180(7) was found to give excellent PA responsiveness to H_2_S within a physiological range from pH 9 to approximately 4.5 (Fig. 3B).

Si@BODPA180(7) also showed a highly selective PA response to H_2_S over various potentially interfering species (Fig. 3C and S6†). It was noticeable that only H_2_S induced a bright PA signal upon excitation at 780 nm, while other analytes, including reactive sulfur (RSS), oxygen (ROS), and nitrogen (RNS) species triggered no obvious PA response. Of note, the strong PA signal elicited by H_2_S was not affected by the presence of 1 mM GSH and 1 mM Cys in competition assays, highlighting the potential applications in a complex biological system.

**Fig. 4:**
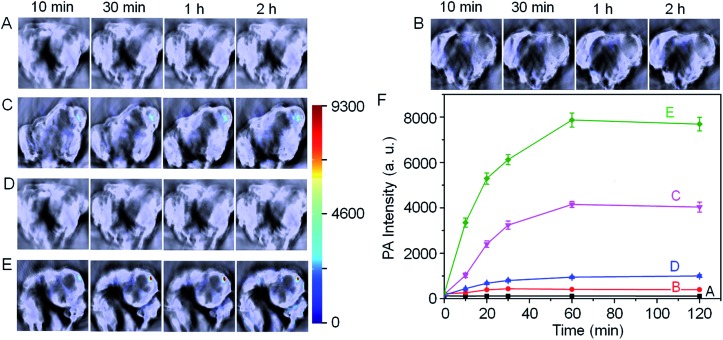
*In vivo* photoacoustic imaging of tumor-bearing mice using Si@BODPA180(7) (10 nmol BODPA1). Saline or the probe in PBS was injected subcutaneously into the tumor regions and normal regions in the dorsal side of living mice, and images were taken at various time points after injection. (A) Saline-treated mice in the tumor regions; (B) probe-treated mice in the normal sites; (C) probe-treated mice in the tumor regions; mice pretreated with (D) 100 nmol AOAA, (E) 300 nmol SAM for 12 h, were subcutaneously injected Si@BODPA180(7) in the tumor regions. (F) PA intensities as a function of time post-injection of Si@BODPA180(7).

With these promising results in hand, we next employed Si@BODPA180(7) for the *in vivo* photoacoustic imaging of endogenous H_2_S generation in a HCT116 tumor-bearing mouse model. It has been suggested that the H_2_S-producing enzyme cystathionine-β-synthase (CBS) is overexpressed in colon cancer, resulting in increased H_2_S production.^13^ In this study, we employed Si@BODPA180(7) as an imaging tool to provide direct evidence for supporting such a functional relationship. In these experiments, saline or Si@BODPA180(7) were injected subcutaneously into tumor regions and normal regions, and images were recorded at various times after probes injection (Fig. 4). To our delight, robust activation of PA signals could be observed specifically in the tumor region in the probe-treated mice within 10 min post-injection (Fig. 4C), indicative of a rapid activation of the probe *in vivo*. Notably, minimal PA signals were observed in the normal site injected with the probe within 2 h (Fig. 4B). Furthermore, no detectable PA signals in the tumor and normal sites could be observed in the saline-treated mice (Fig. 4A). These results indicated that Si@BODPA180(7) can serve as a promising probe for imaging markedly enhanced H_2_S production in colon tumors.

In our study, we also found that the PA signals were greatly attenuated by the CBS inhibitor aminooxyacetic acid (AOAA) (Fig. 4D). In sharp contrast, addition of an allosteric CBS activator *S*-adenosyl-l-methionine (SAM) afforded dramatic elevation of PA signals (Fig. 4E). These preliminary imaging studies suggested that HCT116 colon tumors exhibited CBS upregulation activity which resulted in an increased rate of H_2_S generation. Undoubtedly, this activatable probe can be explored for trapping transient H_2_S and for the real-time monitoring of H_2_S-related biological processes *in vivo*.

## Experimental

### Live subject statement

All animal experiments were performed in compliance with the relevant laws and institutional guidelines for the Care and Use of Research Animals established by Fudan University Animal Studies Committee, and the experiments were approved by the committee.

### General procedure for preparation of Si@BODPAs

BODPAs (0.3 μmol) were rapidly poured into a solution of mPEG-DSPE (19.1 mg) in 3 mL 0.85 N hydrochloride solution under rigorous stirring, resulting in the formation of nanosized micelles. The *in situ* shell cross-linking under acidic conditions with desired amount of silylation agent was carried out at room temperature while kept stirring for 24 h. Then the aqueous solution was dialyzed for 2 days, and the stock solution was thus obtained, which can be diluted to the desired concentration for further studies. The concentration of BODPA1 or BODPA2 in Si@BODPAs was determined according to their standard UV-vis absorption.

The *in situ* shell cross-linking under acidic conditions with TBNBr of 5.73 mg (TBNBr/mPEG-DSPE = 30/100, w/w), 17.19 mg (TBNBr/mPEG-DSPE = 90/100, w/w), 34.38 mg (TBNBr/mPEG-DSPE = 180/100, w/w) and 51.57 mg (TBNBr/mPEG-DSPE = 270/100, w/w), afforded water dispersible nanocomposites Si@BODPA30, Si@BODPA90, Si@BODPA180 and Si@BODPA270, respectively.

BODPA1 (0.3 μmol), mPEG-DSPE (19.1 mg) and TBNBr (34.38 mg) afforded Si@BODPA180(1); BODPA1 (1.2 μmol), mPEG-DSPE (19.1 mg) and TBNBr (34.38 mg) afforded Si@BODPA180(4); BODPA1 (2.1 μmol), mPEG-DSPE (19.1 mg) and TBNBr (34.38 mg) afforded Si@BODPA180(7).

## Conclusions

In summary, we have successfully presented an activatable photoacoustic probe for imaging endogenous H_2_S in living mice. The probe was fabricated by encapsulating semi-cyanine-BODIPY hybrid dyes into the hydrophobic interior of core–shell silica nanocomposites, thus endowing the probe with good water-solubility and excellent biocompatibility. In the presence of H_2_S, a high NIR absorption around 780 nm was triggered, ultimately leading to a strong photoacoustic signal output in the NIR region. Furthermore, the designed probe showed a highly selective PA response to H_2_S with a favourable detection limit of 53 nM. In particular, this probe displayed an extremely fast response, permitting the trapping of transient H_2_S and real-time monitoring of H_2_S-related biological processes. More importantly, this probe could serve as a promising tool for the direct photoacoustic imaging of endogenous H_2_S generation in a HCT116 tumor-bearing mouse model, verifying the activating effect of CBS upregulation on the elevated level of H_2_S. To the best of our knowledge, this work represents the first example of NIR-activatable photoacoustic probes capable of reporting the endogenous production of H_2_S in real time, which may facilitate further exploration of the complex roles of H_2_S in living systems.

## References

[cit1] Kimura H. (2010). Antioxid. Redox Signaling.

[cit2] Tang G., Yang G., Jiang B., Ju Y., Wu L., Wang R. (2013). Antioxid. Redox Signaling.

[cit3] Kamoun P., Belardinelli M.-C., Chabli A., Lallouchi K., Chadefaux-Vekemans B. (2003). Am. J. Med. Genet..

[cit4] Lin V. S., Chen W., Xian M., Chang C. J. (2015). Chem. Soc. Rev..

[cit5] Lippert A. R., New E. J., Chang C. J. (2011). J. Am. Chem. Soc..

[cit6] Liu C., Pan J., Li S., Zhao Y., Wu L. Y., Berkman C. E., Whorton A. R., Xian M. (2011). Angew. Chem., Int. Ed..

[cit7] Sasakura K., Hanaoka K., Shibuya N., Mikami Y., Kimura Y., Komatsu T., Ueno T., Terai T., Kimura H., Nagano T. (2011). J. Am. Chem. Soc..

[cit8] Wang L. V., Hu S. (2012). Science.

[cit9] Pu K., Shuhendler A. J., Jokerst J. V., Mei J., Gambhir S. S., Bao Z., Rao J. (2014). Nat. Nanotechnol..

[cit10] Lovell J. F., Jin C. S., Huynh E., Jin H., Kim C., Rubinstein J. L., Chan W. C. W., Cao W., Wang L. V., Zheng G. (2011). Nat. Mater..

[cit11] Wu X., Chang S., Sun X., Guo Z., Li Y., Tang J., Shen Y., Shi J., Tian H., Zhu W. (2013). Chem. Sci..

[cit12] Bai S., Yang H., Wang P., Gao J., Li B., Yang Q., Li C. (2010). Chem. Commun..

[cit13] Szabo C., Coletta C., Chao C., Módis K., Szczesny B., Papapetropoulos A., Hellmich M. R. (2013). Proc. Natl. Acad. Sci. U. S. A..

