# Supplementary material for: Photoacoustic probes for real-time tracking of endogenous H2S in living mice
Source: Chem Sci. 2016 Nov 30;8(3):2150–5. doi: 10.1039/c6sc04703c (PMC5407269; doi:10.1039/c6sc04703c)
Supplement: Supplementary file 1 [file SC-008-C6SC04703C-s001.pdf]

Electronic Supplementary Material (ESI) for Chemical Science.
This journal is © The Royal Society of Chemistry 2016

Photoacoustic Probes for Real-Time Tracking of Endogenous H_2S in Living Mice

Ben Shi, Xianfeng Gu, Qiang Fei, Chunchang Zhao,*

Key Laboratory for Advanced Materials and Institute of Fine Chemicals, School of Chemistry and Molecular Engineering, East China University of Science & Technology, Shanghai 200237, P. R. China.

Department of Medicinal Chemistry, School of Pharmacy, Fudan University, Shanghai, 201203 China.

1. Synthesis.

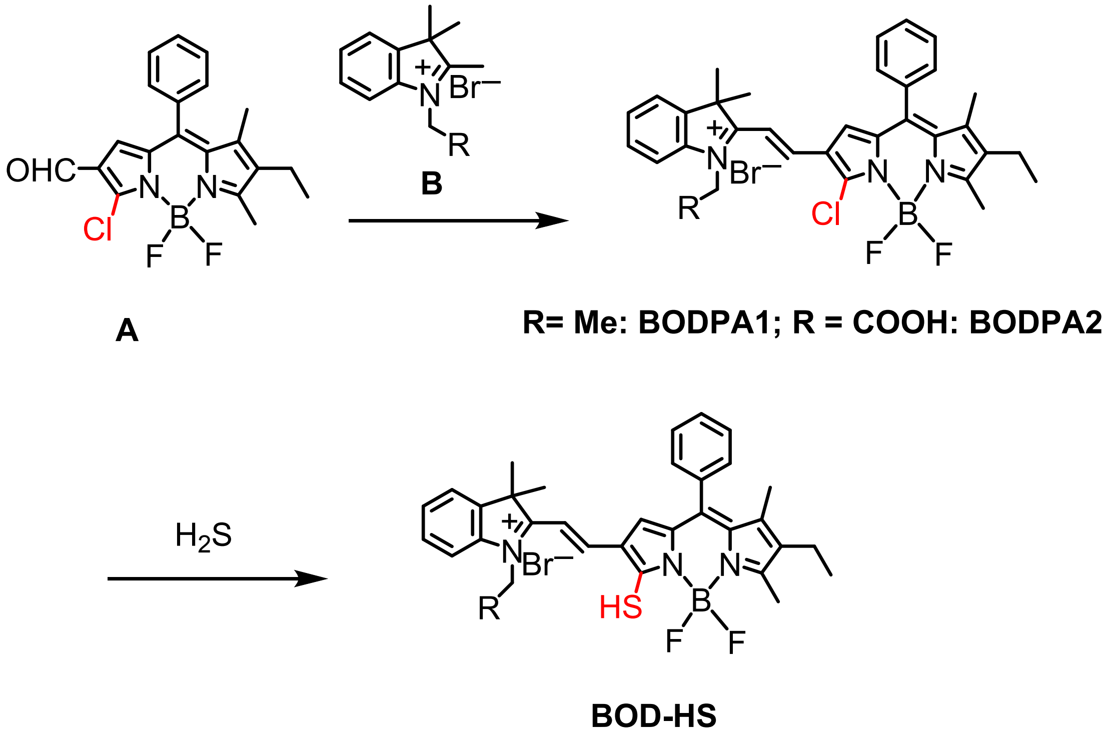

Scheme S1. The synthesis of BODPAs and its reaction with H_2S.

The solution of A (386 mg, 1 mmol) and B (298 mg, 1 mmol; R = COOH) in 30 mL absolute ethanol was refluxed for 5 h under N_2. Subsequently, solvent was removed and the crude was

purified by column to afford 466 mg (70%) BODPA2. ^{1}H NMR (400 MHz, $CDCl_3$): δ 1.05-1.09 (t, *J* = 8 Hz, 3H), 1.52 (s, 3H), 1.78 (s, 6H), 2.41 (q, *J* = 8 Hz, 2H), 2.71 (s, 3H), 4.30 (s, 2H), 7.29 (s, 1H), 7.42-7.44 (m, 2H), 7.49-7.56 (m, 4H), 7.61-7.63 (m, 4H), 8.01-8.05 (d, *J* = 16 Hz, 1H). ^{13}C NMR (100 MHz, $CDCl_3$): δ 12.8, 13.9, 14.1, 17.3, 22.7, 27.5, 31.6, 31.8, 51.8, 110.5, 114.6, 122.0, 122.3, 123.3, 128.9, 129.2, 129.4,129.6, 130.5, 131.8, 135.4, 136.2, 139.3, 140.1, 140.6, 141.6, 142.5, 144.0, 144.6, 168.8, 181.1. HRMS: calculated for $C_{33}H_{32}BClF_2N_3O_2$ [M-Br]$^+$: 585.2280, found: 585.2272.

2. Preparation of Si@BODPAs.

General procedure: BODPAs (0.3 μmol) were rapidly poured into a solution of mPEG-DSPE (19.1 mg) in 3 mL 0.85 N hydrochloride solution under rigorous stirring, resulting in the formation of nanosized micelles. The in-situ shell cross-linking under acidic conditions with desired amount of silylation agents was carried out at room temperature while kept stirring for 24 h. Then the aqueous solution was dialyzed for 2 days, and the stock solution was thus obtained, which can be diluted to the desired concentration for further studies. The concentration of BODPA1 or BODPA2 in Si@BODPAs was determined according to the standard UV-vis absorption.

The in-situ shell cross-linking under acidic conditions with TBNBr of 5.73 mg (TBNBr/mPEG-DSPE = 30/100, w/w), 17.19 mg (TBNBr/mPEG-DSPE = 90/100, w/w), 34.38 mg (TBNBr/mPEG-DSPE = 180/100, w/w) and 51.57 mg (TBNBr/mPEG-DSPE = 270/100, w/w), afforded water dispersible nanocomposites Si@BODPA30, Si@BODPA90, Si@BODPA180 and Si@BODPA270, respectively.

BODPA1 (0.3 μmol), mPEG-DSPE (19.1 mg) and TBNBr (34.38 mg) afforded Si@BODPA180(*1*);

BODPA1 (1.2 μmol), mPEG-DSPE (19.1 mg) and TBNBr (34.38 mg) afforded Si@BODPA180(*4*);

BODPA1 (2.1 μmol), mPEG-DSPE (19.1 mg) and TBNBr (34.38 mg) afforded Si@BODPA180(*7*).

3. *In vivo* imaging.

HCT116 cells (2×10^6) suspended in 200 μl of PBS were subcutaneously injected at the dorsal side location in female nude mice. 3 weeks after implantation, Saline or Si@BODPA180(*7*) (10 nmol BODPA1) in PBS at a total volume of 200 μl was injected subcutaneously into tumor and normal regions in HCT116 tumor-bearing mice. Photoacoustic images for mice anesthetized with isoflurane were recorded at various times after probe injection using MSOT inVision 128, iThera Medical Germany.

4. HPLC and HRMS were utilized for monitoring the reaction of BODPA2 +NaHS.

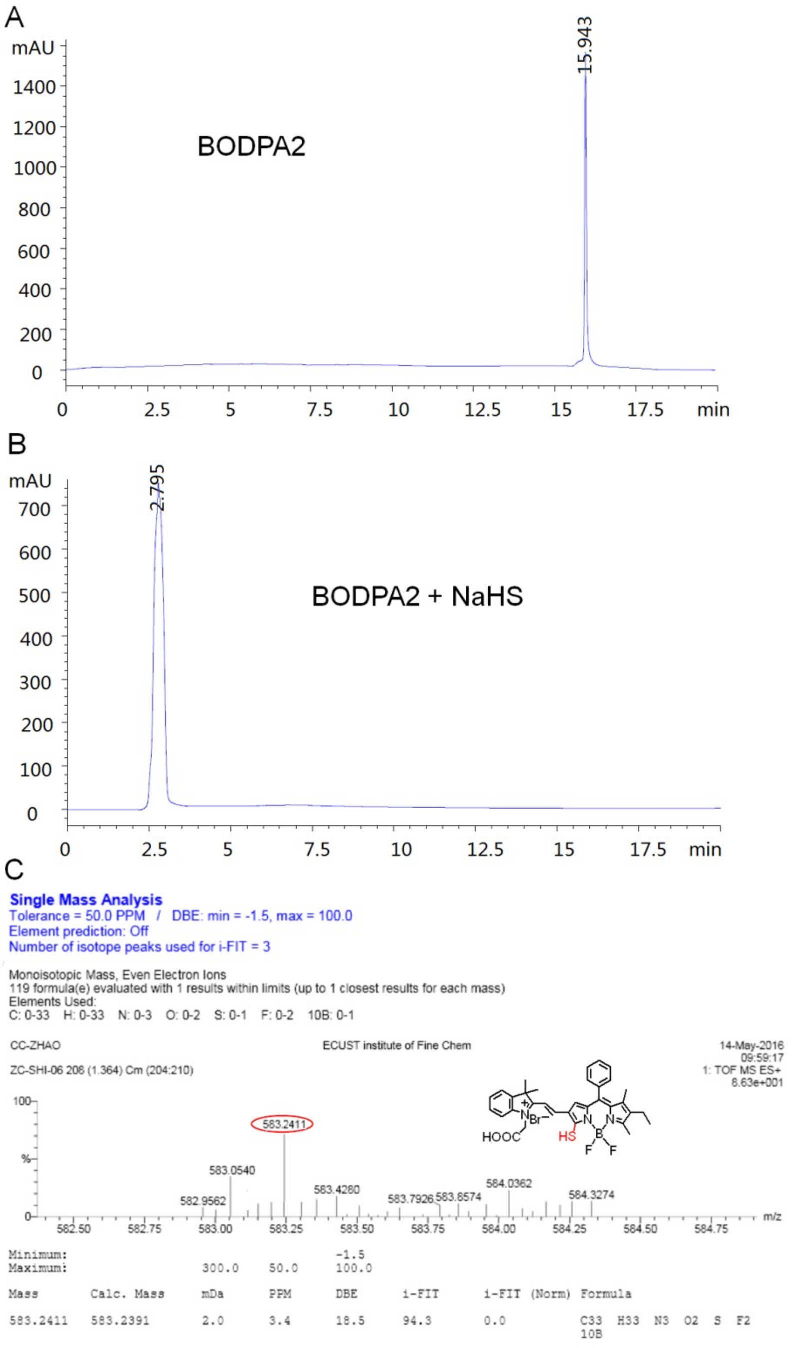

Fig. S1 HPLC profiles of BODPA2 (A) and BODPA2 +NaHS (B). (C) HRMS spectrum of BODPA2 +NaHS. BODPA2 (5 μM, HPLC retention time at 15.9 minutes and m/z at 585.2272) proceeded S_NAr with H_2S and was efficiently converted to sulfide-substituted BODIPY (HPLC retention time at 2.8 minutes and m/z at 583.2411).

5. Time-dependent spectra changes of BODPAs in the presence of NaHS.

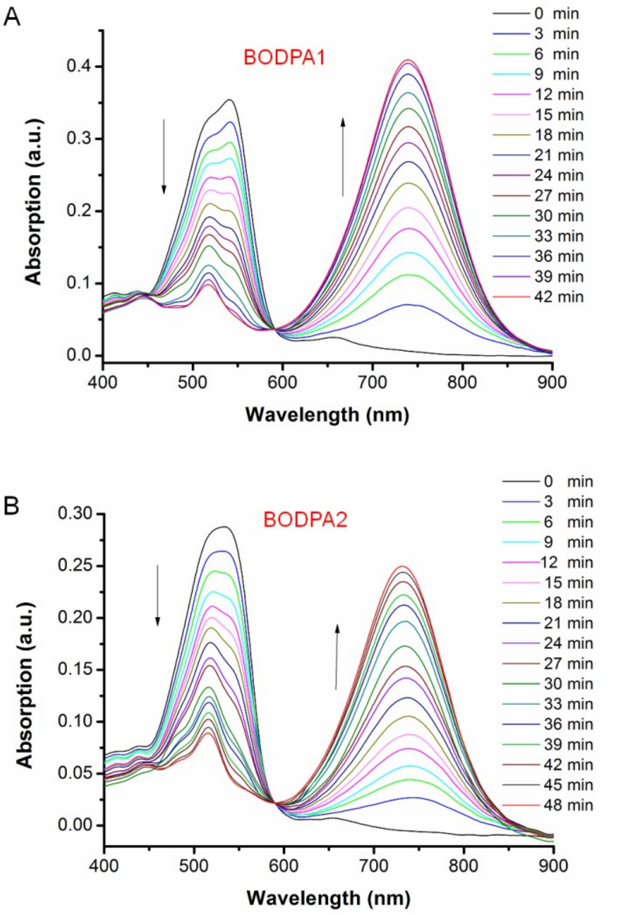

Fig. S2 Time-dependent absorption spectra changes of (A) BODPA1 (10 μM) and (B) BODPA2 (10 μM) in the presence of NaHS (100 μM) under model conditions (acetonitrile/PBS buffer, 1:1, v/v, 20 mM, pH 7.4, room temperature).

6. DLS and TEM characterizations.

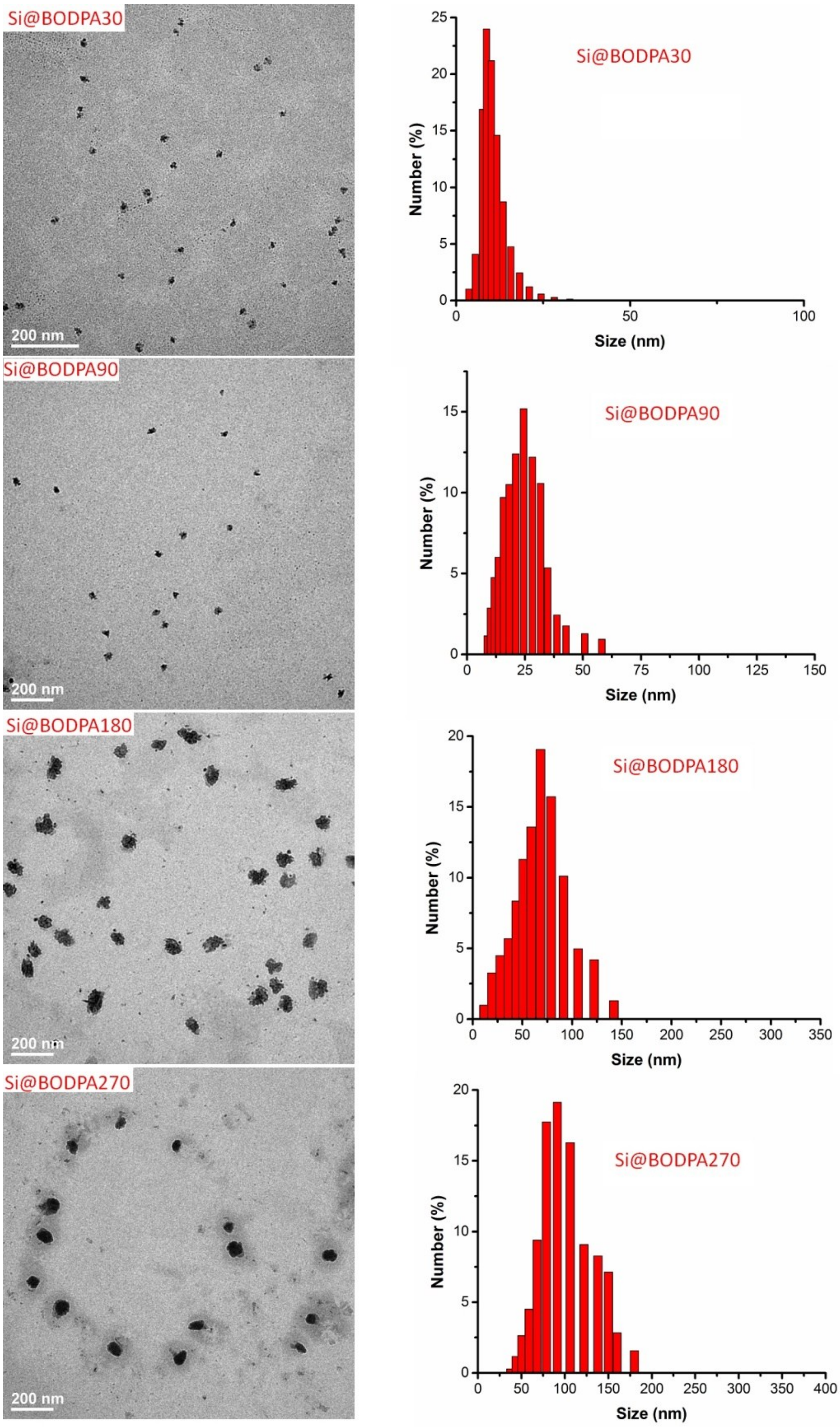

Fig. S3 Dynamic light scattering and TEM measurements for evaluation of the average size of Si@BODPAs.

7. Time-dependent spectra changes of Si@BODPAs in the presence of NaHS.

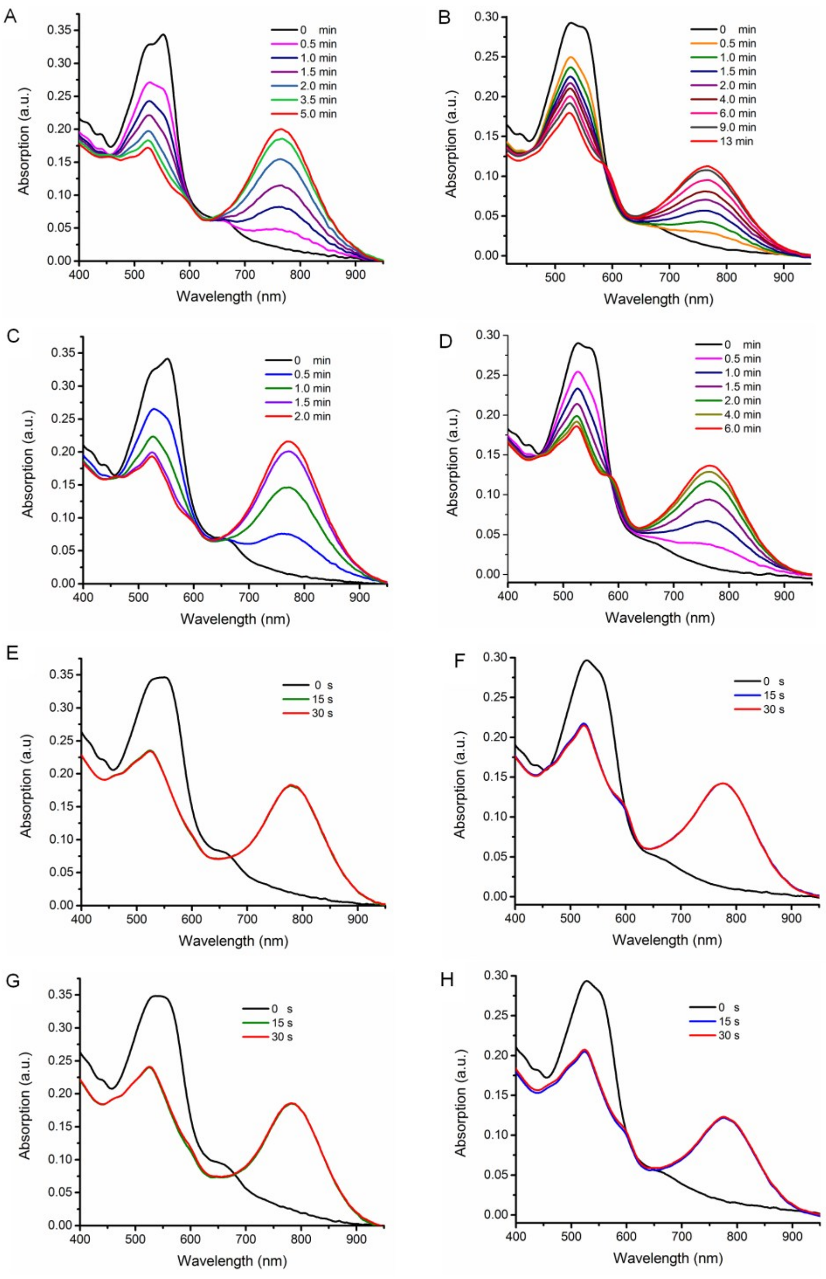

Fig. S4 Time-dependent absorption spectra changes of (A) Si@BODPA30 (10 μM BODPA1), (B) Si@BODPA30 (10 μM BODPA2), (C) Si@BODPA90 (10 μM BODPA1), (D) Si@BODPA90 (10 μM BODPA2), (E) Si@BODPA180 (10 μM BODPA1), (F) Si@BODPA180 (10 μM BODPA2), (G) Si@BODPA270 (10 μM BODPA1) and (H) Si@BODPA270 (10 μM BODPA2) in the presence of NaHS (100 μM) in PBS (pH 7.4) at room temperature.

8. EDX characterizations.

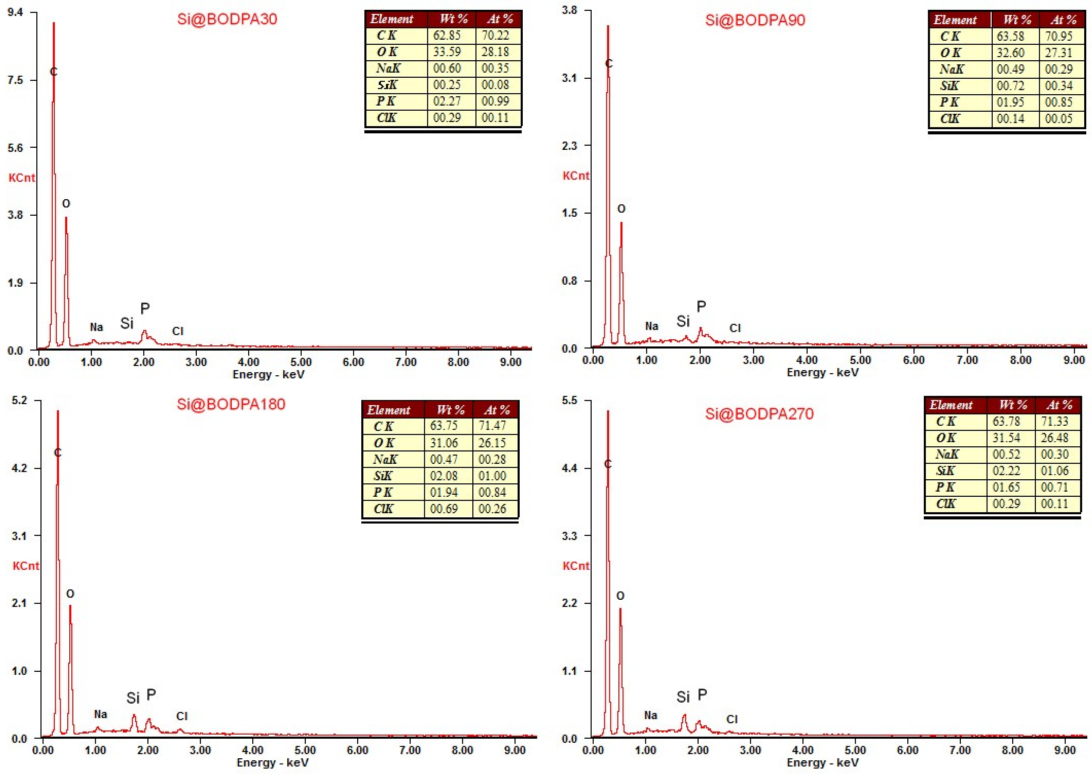

Fig. S5 The energy dispersive X-ray spectrum for demonstrating the existence of silica shell.

9. The selectivity of Si@BODPA180(*7*) to H_2S over related analytes.

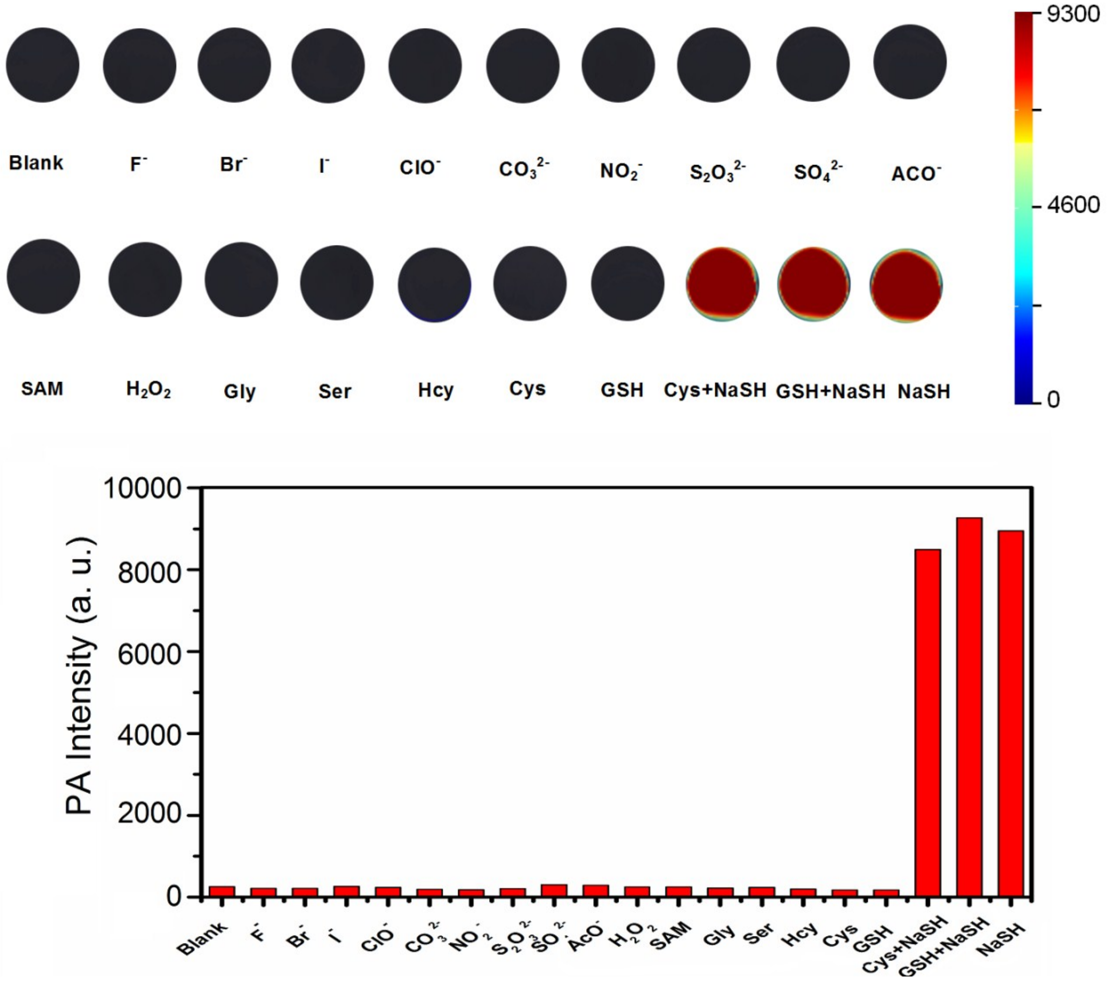

Fig. S6 PA responses of Si@BODPA180(*7*) to 100 μM NaHS and other biologically relevant competing analytes in PBS (pH 7.4) at room temperature. Data shown are for 1 mM glutathione, 1 mM cysteine, and 100 μM for other analytes. It should be mentioned that GSH and Cys had minimal influence on the PA responsiveness. SAM: S-adenosyl-L-methionine.

10. NMR and HRMS characterization.

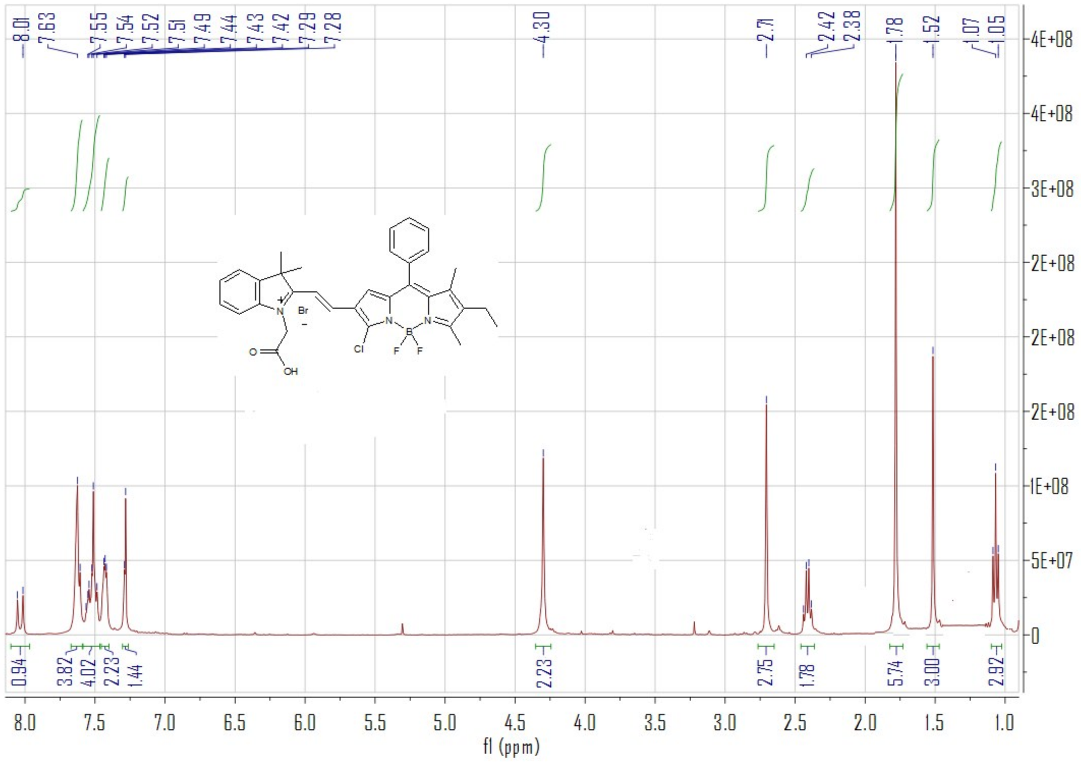

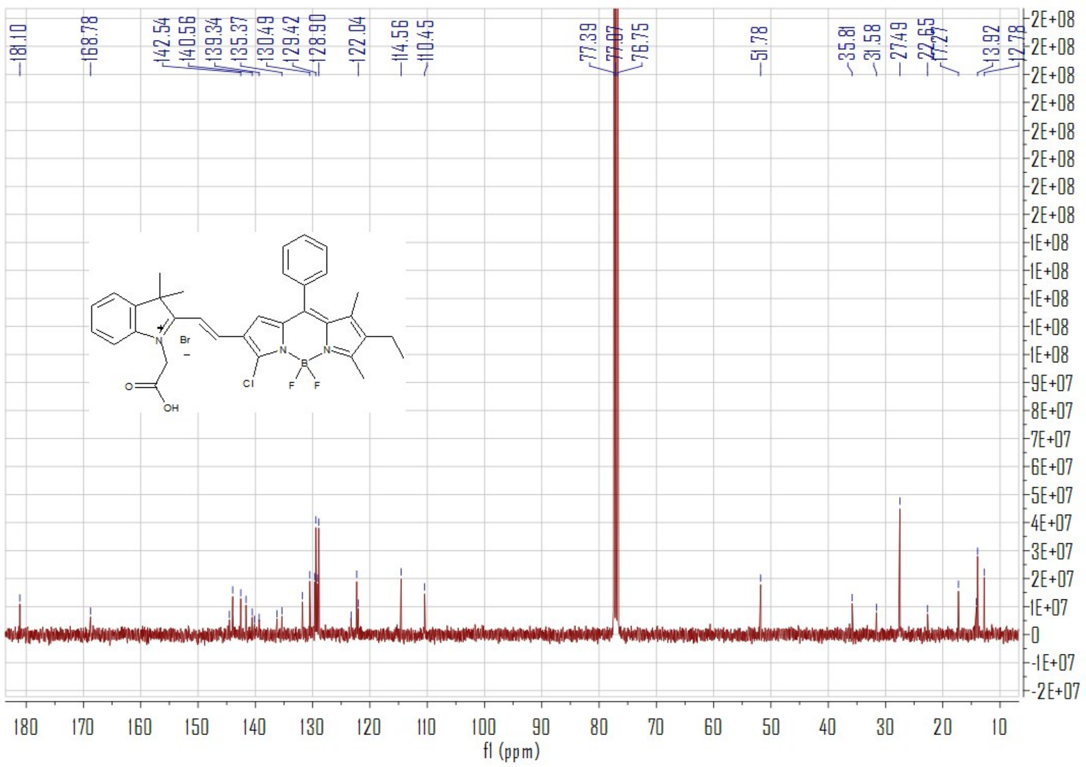

Single Mass Analysis

Tolerance = 50.0 PPM / DBE: min = -1.5, max = 100.0
Element prediction: Off
Number of isotope peaks used for i-FIT = 3

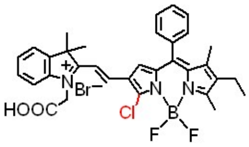

Monoisotopic Mass, Even Electron Ions
50 formula(e) evaluated with 1 results within limits (up to 1 closest results for each mass)
Elements Used:
C: 0-33 H: 0-40 N: 0-3 O: 0-2 Cl: 1-1 F: 0-2 10B: 0-1

CC-ZHAO ECUST institute of Fine Chem 08-Apr-2016 22:49:25

ZC-SHI-04 153 (1.031) Cm (148:154) 1: TOF MS ES+ 3.08e+002

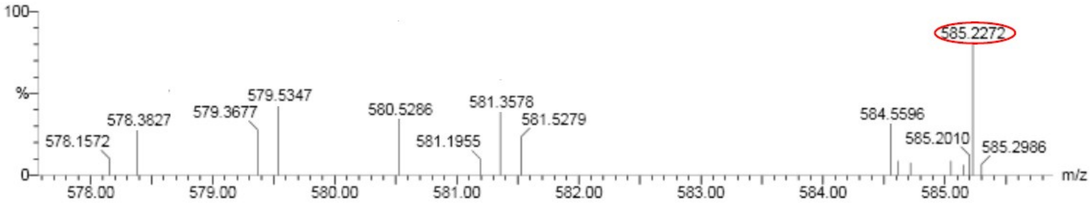

Minimum:				-1.5			
Maximum:		300.0	50.0	100.0			
Mass	Calc. Mass	mDa	PPM	DBE	i-FIT	i-FIT (Norm)	Formula
585.2272	585.2280	-0.8	-1.4	18.5	27.3	0.0	C33 H32 N3 O2 Cl F2 10B
